# Review of Life-Cycle Approaches Coupled with Data Envelopment Analysis: Launching the CFP + DEA Method for Energy Policy Making

**DOI:** 10.1155/2015/813921

**Published:** 2015-01-12

**Authors:** Ian Vázquez-Rowe, Diego Iribarren

**Affiliations:** ^1^Public Research Centre Henri Tudor (CRPHT)/Resource Centre for Environmental Technologies (CRTE), 6A Avenue des Hauts-Fourneaux, 4362 Esch-sur-Alzette, Luxembourg; ^2^Peruvian LCA Network, Faculty Department of Engineering, Pontificia Universidad Católica del Perú, 1801 Avenida Universitaria, San Miguel, Lima 32, Peru; ^3^Department of Chemical Engineering, University of Santiago de Compostela, Rúa Lope Gómez de Marzoa s/n, 15782 Santiago de Compostela, Spain; ^4^Instituto IMDEA Energía, 3 Avenida Ramón de la Sagra, 28935 Móstoles, Spain

## Abstract

Life-cycle (LC) approaches play a significant role in energy policy making to determine the environmental impacts associated with the choice of energy source. Data envelopment analysis (DEA) can be combined with LC approaches to provide quantitative benchmarks that orientate the performance of energy systems towards environmental sustainability, with different implications depending on the selected LC + DEA method. The present paper examines currently available LC + DEA methods and develops a novel method combining carbon footprinting (CFP) and DEA. Thus, the CFP + DEA method is proposed, a five-step structure including data collection for multiple homogenous entities, calculation of target operating points, evaluation of current and target carbon footprints, and result interpretation. As the current context for energy policy implies an anthropocentric perspective with focus on the global warming impact of energy systems, the CFP + DEA method is foreseen to be the most consistent LC + DEA approach to provide benchmarks for energy policy making. The fact that this method relies on the definition of operating points with optimised resource intensity helps to moderate the concerns about the omission of other environmental impacts. Moreover, the CFP + DEA method benefits from CFP specifications in terms of flexibility, understanding, and reporting.

## 1. Introduction

The energy sector currently faces severe concerns about energy security and environmental sustainability [1, 2]. However, these two main drivers influencing policies and regulations in the energy sector have shown to provide diverging recommendations for future energy strategies [3]. Nevertheless, most energy observation organisations, such as the International Energy Agency (IEA) or the US Energy Information Administration (US-EIA), agree in stating that if current energy policies do not experiment clear changes by 2020, imports of fossil fuels in most developed and emerging nations, as well as the derived greenhouse gas (GHG) emissions, will continue to soar [4]. In fact, a growing number of scientists are starting to consider that the pace of implementation for higher energy efficiency standards and decarbonised energy sources is currently unable to quench the increasing thirst for energy by human populations [5].

Increasing social awareness on these issues has fostered the research and development of clean and renewable energy systems, as well as the establishment of management schemes to evaluate and improve their environmental performance. According to this socioeconomic context, current energy policies are focused on the promotion of environmentally sustainable energy systems. For instance, at a European level the Renewable Energy Directive 2009/28/EC [6] and related documents [7] provide the policy framework for developing a low-carbon energy system.

Current energy policies tend to identify environmentally sustainable energy with low-carbon energy, limiting the environmental dimension of the energy system to its global warming impact. This fact responds to the recognized need to act on GHG emissions to mitigate climate change, being, therefore, the major socioenvironmental concern to be addressed by energy actors through the whole supply chain [8, 9]. In fact, given that the energy sector is the worldwide economic activity with a higher contribution to GHG emissions (26%), the objectives of reaching a 450 ppm CO_2_ atmospheric concentration stabilisation target by 2050 are highly bound to the enhancement of energy efficiency and the minimization of fossil fuel use in this sector [10, 11].

Working at different scales and with interdisciplinary approaches is needed to enhance cleaner production alternatives and technological development, enabling the energy sector to meet the established objectives [8, 12]. For instance, analytical methods to verify the suitability of the corrective actions implemented in the energy sector are needed. Consequently, the development of specific modelling tools to understand the interactions between the main energy policy drivers is currently being promoted [13, 14].

The environmental sustainability of an energy system is usually measured in terms of the associated savings in GHG emissions. These GHG savings are calculated based on the global warming impact potential (GWP) of the assessed energy system in comparison with that of the conventional fossil equivalent, following a life cycle (LC) perspective [6]. Due to the need for quantifying the LC-GHG emissions of product systems (need not be restricted to the energy sector), several carbon footprinting (CFP) specifications have arisen in recent years, PAS 2050:2011 [15] and the Greenhouse Gas Protocol [16] being some of the most relevant proposals together with the recent ISO/TS 14067:2013 [17].

The completeness of CFP in terms of methodological soundness makes it comparable to the well-established life cycle assessment (LCA) methodology [18, 19]. In fact, CFP can be considered, not exempt from certain nuances, an LCA limited to the GWP impact category [20, 21]. Not surprisingly, LCA is the most common tool for quantifying GHG emissions [22] although its holistic and comprehensive perspective allows covering a wider range of environmental impacts (e.g., resources depletion, damage to the ecosystem, toxicities, acidification, etc.).

LC-based methodologies are of paramount importance in energy policy making, as they arise as a relevant source of criteria to be taken into account when it comes to defining policies and regulations. In particular, these policies often have to state quantitative benchmarks that orientate the performance of specific energy systems towards environmental sustainability. Robust LC-based methodologies that facilitate the quantification of these benchmarks are therefore required. However, while LC-based methods are highly used to understand the relevant sources of environmental impacts within the LC of the assessed system, their utility as single methodologies for ecoefficiency verification and environmental impact minimisation across complex sectoral systems is frail.

In this context, data envelopment analysis (DEA) arises as a linear programming methodology to measure the relative efficiency of multiple homogenous entities when the productive process shows a structure composed of multiple inputs and outputs [23]. DEA also allows the quantification of target feasible operating conditions that would turn inefficient entities into comparatively efficient ones, thus arising as a valuable tool for benchmarking purposes.

DEA is being increasingly combined with LC approaches to provide LC-based benchmarks. The resultant combined methodologies are of general use and they are only conditioned by the availability of input and output data for a set of multiple homogenous entities, normally called decision-making units (DMUs) [24]. Within this framework, the LCA + DEA methodology was formally presented in 2010 as a combination of LCA and DEA to benchmark the operational and environmental performance of resembling entities [25, 26]. Even though LCA + DEA studies to date have mainly assessed agrifood systems, the LCA + DEA methodology can be applied to any type of sector [24]. For instance, Iribarren et al. [27] have recently carried out the LCA + DEA study of wind farms, showing that this methodology can be useful for the benchmarking of energy conversion systems.

Beyond the combined use of LCA and DEA, other LC-based methodologies have been coupled with DEA, leading to the breakthrough of the LC + DEA concept. For example, emergy analysis can be applied in combination with DEA to provide an ecocentric benchmarking tool [28]. However, different implications are associated with each specific LC + DEA method selected for the supply of benchmarks for energy policy making. The present paper thoroughly examines the LC + DEA methods currently available for the provision of benchmarks to policy makers. Furthermore, given the high level of correlation that has been observed between GHG reductions (i.e., environmental improvements) and energy security [4, 14], which leads to the current relevance of GHG emissions mitigation in current energy policies, a novel methodological framework is presented based on the combined use of CFP and DEA as a way to evaluate and benchmark ecoefficiency in the energy sector.

## 2. Review of LC + DEA Methods

**Figure 1:**
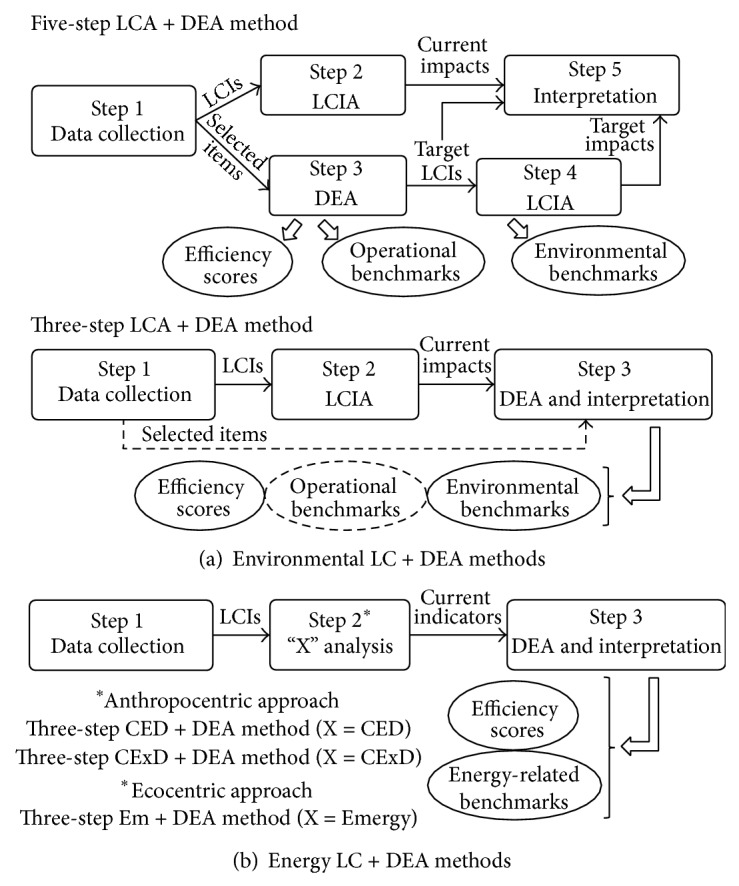
LC + DEA methods currently available (CED: cumulative energy demand; CExD: cumulative exergy demand; DEA: data envelopment analysis; Em: emergy; LCA: life cycle assessment; LCI: life cycle inventory; LCIA: life cycle impact assessment).

Current LC + DEA methods for the benchmarking of multiple DMUs can be divided into two main blocks: those that are inspired on the direct monitoring of environmental benchmarks and those that assess these benchmarks through the computation of energy methods. In other words, the former focus on the benchmarking of environmental indicators, while the latter provide benchmarks expressed in energy terms.  Figure 1 shows the key methodological steps of the available methods.

### 2.1. Environmental LC + DEA Methods

To date, two specific methods have been developed regarding environmental LC + DEA methods. On the one hand, the five-step LCA + DEA method leads to the calculation of environmental benchmarks directly associated with the optimised operational performance of the DMUs [29]. This requires (i) data collection to define the life cycle inventory (LCI) of each of the DMUs, (ii) the subsequent life cycle impact assessment (LCIA) to determine their current environmental profiles, (iii) a DEA study to identify efficient DMUs and calculate target operating points (i.e., operational benchmarks) for the inefficient entities, (iv) a new LCIA taking into account the target DMUs in order to calculate the corresponding environmental benchmarks, and (v) result interpretation for ecoefficiency verification [26]. Furthermore, this five-step method has been recently proposed as an easy-to-implement concept oriented towards sustainability assessment when integrating socioeconomic parameters into the analysis [30].

On the other hand, the three-step LCA + DEA method addresses the direct benchmarking of the environmental impacts of the DMUs under assessment, also allowing the simultaneous benchmarking of operational items [31]. The data collection and LCIA steps of this method are analogous to those of the five-step method. However, in the three-step LCA + DEA method, the environmental profiles coming from the LCIA phase are directly fed to the DEA step, thus obtaining the environmental benchmarks without requiring a second LCIA. Although this alternative leads to a relatively rapid environmental benchmarking, it has been mainly regarded as a preliminary assessment due to consistency issues linked to the lack of independence between DEA inputs [24].

### 2.2. Energy LC + DEA Methods

Regarding the available energy LC + DEA methods, three different perspectives have been developed, each of which with distinct features due to the different indicators subject to benchmarking. These methods can be classified according to their anthropocentric or ecocentric perspective.

On the one hand, anthropocentric alternatives include the three-step CED + DEA and CExD + DEA methods, which provide benchmarks of cumulative energy demand (CED) and cumulative exergy demand (CExD) indicators, respectively [28]. On the other hand, the three-step Em + DEA method—based on the emergy (Em) concept—is an available option for ecocentric benchmarking [28]. All these energy LC + DEA methods involve similar data collection and DEA steps, but they differ in the technique used to analyse LCI data in energy terms.

### 2.3. Overview of LC + DEA Case Studies

A survey of the main LC + DEA case studies conducted to date is presented in Table 1. As shown in this table, the vast majority of the studies use environmental LC + DEA methods. This observation is closely linked to the novelty (i.e., recent development) of the energy LC + DEA methods, which account for only one case study [28].

The five-step LCA + DEA method is found to be the methodological approach most often selected by LC + DEA practitioners. To date, this approach has been mainly applied to the primary sector, for example, for agriculture [32–35], aquaculture [29], and fishing [26, 30, 36, 37]. Furthermore, the suitability of this approach for application in other sectors (e.g., the energy sector) has already been proved [27]. Alternatively, the use of life cycle environmental indicators as DEA inputs in three-step approaches also accounts for a relevant number of case studies [31, 38–42].

### 2.4. Perspectives

Given the novelty of the LC + DEA concept, it is expected that new LC + DEA methods may emerge in the future as a result of further research in the fields of environmental management and ecoefficiency. Furthermore, the expected methodological development of social life cycle assessment (SLCA) and life cycle sustainability assessment (LCSA) may provide interesting insights into the use of LC + DEA methods as related to the social and economic pillars of sustainability [30, 43, 44]. In contrast, other available LC + DEA strategies can be seen as variants of the aforementioned methods. For instance, the three-step LCA + DEA method can include the use of hybrid LCA approaches such as economic input-output LCA [45]. Finally, some existing DEA studies optimise environmental indicators and/or environmental burdens, but the involved methods cannot be classified as LC + DEA since the utilised environmental parameters do not show an LC perspective [46–49].

A comprehensive evaluation of environmental impact categories (in LCA studies) and/or energy indicators (in CED, CExD, and Em analyses), while providing a solid basis for the determination of a thorough set of benchmarks useful for (energy) policy making, can hinder a single interpretation of the results/benchmarks. When there is a special interest in specific environmental or energy indicators, more straightforward LC + DEA approaches could be conceived. In particular, since energy policies focus on GWP and diverse schemes for the calculation of LC-GHG emissions are available or in progress, a combined method based on CFP and DEA is proposed in this paper: the CFP + DEA method (Section 3).

## 3. The CFP + DEA Method

**Figure 2:**
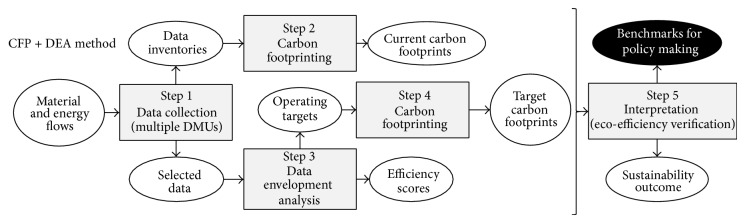
The five-step CFP + DEA method.

A five-step method combining CFP and DEA is herein proposed to provide benchmarks for energy policy making by (i) collecting data on the material and energy flows of multiple DMUs, (ii) evaluating the carbon footprint of each current DMU, (iii) benchmarking the operational performance of the DMUs through DEA, (iv) evaluating the carbon footprints of virtual DMUs that incorporate the operational benchmarks, and (v) interpreting the results to provide environmental benchmarks for policy making and additional outcome (Figure 2). A three-step method for direct benchmarking of current carbon footprints via DEA is also feasible, but this option is ruled out here due to the fact that it does not allow analysts to link CFP benchmarks and target operating points (in other words, the identification of the sources of inefficiency is impeded).

As can be observed by comparing Figures 1 and 2, the proposed CFP + DEA method involves a similar procedure to that of the five-step LCA + DEA method. However, it should be noted that, in the CFP + DEA method, the CFP steps (i.e., steps 2 and 4 in Figure 2) may consist of either LCA studies restricted to the GWP category or alternative analytical schemes to calculate LC-GHG emissions (e.g., according to available technical specifications such as PAS 2050:2011 [15] and ISO/TS 14067:2013 [17]). Special features of the CFP + DEA method are discussed later in Section 4.

The final step of the CFP + DEA method which deals with the interpretation of the results from the previous steps merits further attention. In particular, the target carbon footprints from step 4 are fed to the fifth step to define the environmental benchmarks needed for energy policy making. Individual benchmarks may be necessary to make policies aimed at a specific set of entities. In other cases, overall benchmarks (based on efficient DMUs) are needed to set general objectives for entities at a larger scale. In those cases in which a large number of evaluated DMUs are found to operate efficiently but the use of a reduced set of CFP benchmarks drawn from the best-performing units is intended for policy making, the complementary use of superefficiency DEA models to discriminate among efficient entities is encouraged [24, 50].

Additional outcome from the final step of the CFP + DEA method includes ecoefficiency verification and the possibility to discuss environmental, economic, and social aspects under the same framework for sustainability assessment [30]. According to the traditional concept of ecoefficiency [51] and taking into account the limitation of the CFP + DEA method to GWP, ecoefficiency verification is understood as the quantitative proof that the delivery of goods with reduced resource intensity leads to lower impacts in terms of GWP.

## 4. Discussion

### 4.1. Screening of LC + DEA Methods for Energy Policy Making

**Figure 3:**
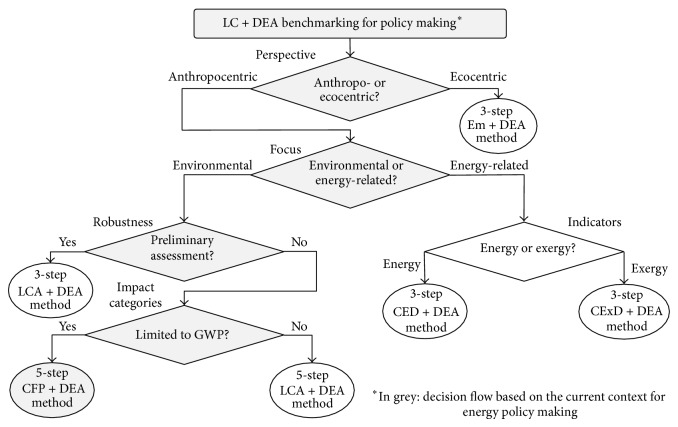
Decision flowchart to select the most appropriate LC + DEA method for policy making according to current methodological options (including the CFP + DEA method).

All the LC + DEA methods mentioned in this paper allow benchmarking and have the potential of being used for policy making. However, each method involves different benchmarking perspectives and/or focuses on different indicators as benchmarking criteria. A decision flowchart to facilitate the appropriate selection of a specific LC + DEA method for policy making is proposed in Figure 3. In addition to currently available LC + DEA methods (i.e., five- and three-step LCA + DEA methods and three-step CED + DEA, CExD + DEA, and Em + DEA methods), the five-step CFP + DEA method is also taken into account.

As the current context for energy policy making implies an anthropocentric perspective with focus on the environmental concerns, and specifically on GWP, the five-step CFP + DEA method is expected to be the most consistent LC + DEA approach for the provision of benchmarks with energy policy making purposes.

### 4.2. Adequacy of Implementing CFP + DEA Case Studies in the Energy Sector

The reduction in anthropogenic GHG emissions has become a key issue in international environmental policy due to its relevance when it comes to mitigating climate change [52]. However, the single use of specific indicators and methods (e.g., CFP) to monitor GHG emissions, overlooking other environmental dimensions, may seem a myopic approach towards sustainable development [21]. This perspective has been defended in the literature [53], based on the weak correlation between CFP and certain impact categories (e.g., human toxicity, ecotoxicity, etc.). Despite this limitation, which may derive in misleading environmental assessments in local and regional evaluations, its validity as sole indicator is still very high from a worldwide perspective, given the strong interactions that climate change shows with other global environmental requirements [8, 54], such as ocean acidification, agricultural land use, or biodiversity loss [55, 56]. In other words, global scale reductions of GHG emissions will ultimately trigger reductions in the environmental impact of most of the* planetary boundaries* [54], contributing to fit anthropogenic activities within the Earth's carrying capacity [57].

While reductions in GHG emissions may be attained through multiple schemes, a key strategy in developed countries is the implementation of* power down* policies [8, 58], aiming at reducing energy demand through achieving higher efficiency in technologies and changes in behavioural patterns. For instance, the recently published* Zero Carbon Britain* report [8] suggests that efficiency and changes in behaviour can reduce by 60% in 2030 the GHG emissions linked to three key sectors: buildings, transport, and energy. The decentralised characteristics of these three sectors, as well as the intended strategy to enhance decentralised renewable energy planning, denote the need to develop adequate benchmarks to identify best-performing technologies for energy production and consumption [59, 60]. Therefore, given the higher dispersal in the management of energy, with multiple similar units disseminated throughout the territory to feed the particular needs of local and regional communities, the use of CFP + DEA arises as a convenient methodology to identify sources of inefficiency in multiple energy-related sectors and to highlight the environmental benefits in terms of GHG emissions reductions that may be attained through the minimisation of operational inputs. Furthermore, the CFP + DEA method provides an ideal framework for the identification of strategies to attain GHG emissions reductions through improving the ecoefficiency of energy-related technologies.

### 4.3. Can the CFP + DEA Method Benefit from Existing CFP Specifications?

LCA-related assessment tools, such as CFP, have shown to be fairly flexible in terms of their methodological implementation for two main reasons. On the one hand, these methods are designed to be used worldwide to address the environmental profile of any product or service. Therefore, these methods need sufficient flexibility to adapt to the specific research questions that arise in site- and product-specific situations.

On the other hand, as highlighted by Finkbeiner [61], there are many methodological challenges still to tackle in order to guarantee the completeness of the assessment method. This has led LCA practitioners to approach case studies in many different ways regarding controversial methodological assumptions, such as system boundaries delimitation, allocation procedures, the computation of land use changes, data quality, or the inclusion of capital goods [61]. In addition, there is still no agreement on whether CFP should report all GHG emissions (IPCC perspective), seeking completeness and accuracy in the communication of the results, or only those monitored by the Kyoto Protocol, allowing a more pragmatic GHG reduction strategy according to policy making schemes [61]. When integrating CFP into LC + DEA thinking, the flexibility of the CFP method should be maintained, since it is not the objective of this method to answer highly specific challenges of the methods it combines.

The proliferation of CFP standards fostered by different standardisation organisations (e.g., PAS 2050:2011 [15] and ISO/TS 14067:2013 [17]) may provide an interesting framework for facilitating the development of CFP + DEA case studies. For instance, the recommendations of the supplementary requirements for the application of PAS 2050:2011 to specific productive sectors (e.g., for the horticulture and seafood sectors) requiring to base CFP on a representative sample may constitute an opportunity to implement solid CFP + DEA studies [62, 63]. In addition, the existence of other specifications linked directly to the energy and building sectors, such as PAS 2060:2010 [64] or PAS 2030:2012 [65], may also provide guidance regarding the specific measures to tackle operational inefficiencies.

### 4.4. Strengths and Limitations of the CFP + DEA Method

The main strengths and weaknesses of using the CFP + DEA method for benchmarking and ecoefficiency purposes are summarised in Table 2. The CFP + DEA method, the same as the five-step LCA + DEA method and the energy LC + DEA methods, constitutes a consistent methodological scheme for the evaluation and benchmarking of a set of multiple DMUs, in contrast to the three-step LCA + DEA method [42], which can be considered a preliminary assessment method [24]. However, unlike energy LC + DEA methods, the CFP + DEA and five-step LCA + DEA approaches allow quantifying the specific optimisation of operational inputs needed to ensure the neutralisation of observed inefficiencies throughout the selected sample [28].

The identification of the underlying factors influencing the inefficiency in CFP + DEA studies, in contrast, may not always be achievable, given the multiple factors that may be influencing the performance of a particular DMU [49]. Furthermore, this constraint may also have important consequences from an economic perspective, since the economic costs to attain the desired benchmarks cannot be fully estimated. Nevertheless, a recent EIO-LCA + DEA study suggests the computation of data perturbations in the DEA matrix to identify the relative sensitivity of each input on the final efficiency value [45]. This procedure, while useful for prioritising which strategies will lead to higher quotas of efficiency for inefficient DMUs, still lacks enough disaggregation to determine which are the specific factors influencing the inefficiencies in each specific entity.

A further limitation that has been observed in LC + DEA methods is the limited number of items that can be processed in the DEA matrix [23]. However, in contrast to what has been argued in previous studies [42], this limitation is extensible to any DEA-based approach due to the codependency between the number of DEA items and DMUs that can be assessed, independently of the nature of these items (e.g., operational inputs and/or environmental impact categories in the three-step LCA + DEA method or operational inputs exclusively in five-step methods).

In general, the strengths and limitations that are usually attributed to CFP as compared to regular LCA could be also highlighted for the CFP + DEA method. On the one hand, regarding its strengths, the proliferation of CFP as compared to more comprehensive environmental assessment methods has arisen from the increased interest of stakeholders in understanding and communicating to consumers the LC-GHG emissions linked to their products [61, 66]. Therefore, while the five-step LCA + DEA method can be considered a more comprehensive approach to environmental benchmarking, since the environmental benefits are evaluated for a wider range of environmental dimensions, the CFP + DEA approach provides an easy-to-understand (and report) causeway for the use and communication of environmental benchmarks. In fact, the development of a CFP + DEA method, as suggested in this paper, may allow life cycle thinking to penetrate in decision contexts which regular LCA + DEA methods were unable to reach due to complexity [21, 61], increasing, therefore, the relevance of the methodology.

On the other hand, the main concern about the CFP + DEA method, referring to the limited scope of the environmental assessment in terms of evaluated impacts [21, 53], may not be necessarily seen as a hurdle to the use of the method for energy policy making due to the relevance of GWP in energy policy and to the high interconnections that this environmental dimension has with other major environmental issues. Furthermore, despite the reiterated statement that CFP cannot account for a comprehensive assessment of environmental impacts [53], due to its focus on GHG emissions, this limitation may not be as crucial when applying CFP + DEA. The rationale behind this perspective is linked to the underlying nature of the method, which seeks ultimately environmental benchmarking through the minimisation of operational inputs (i.e., optimisation of resource use). Consequently, reductions in resource use linked to decreasing GHG emissions for the assessed units are bound to generate different levels of environmental benchmarking for other impact categories (e.g., toxicity, acidification, etc.), as already proven in prior LC + DEA studies [32, 33, 45].

## 5. Conclusions and Perspectives

The use of LC + DEA methods to provide benchmarks for energy policy making is feasible provided that inventory data can be collected for multiple homogenous entities. Furthermore, under the current context for energy policies, characterised by an anthropocentric perspective with focus on the mitigation of GHG emissions, the combined use of CFP and DEA is presented as a sector-specific approach to assist energy planners and policy makers to avoid potential energy system bottlenecks. In particular, a five-step CFP + DEA method based on data collection, calculation of target operating points, evaluation of current and target carbon footprints, and result interpretation is developed as a recommended tool for providing policy makers with CFP benchmarks.

Despite its limited scope in terms of environmental impacts (due to the restriction to GWP), the CFP + DEA method constitutes a robust and consistent scheme for the provision of benchmarks for energy policy making. Moreover, the fact that this method relies on the definition of operating points with optimised resource intensity helps to moderate the concerns about the omission of other environmental impacts. Additionally, the CFP + DEA method benefits from CFP specifications in terms of flexibility, understanding, and reporting. Finally, although the CFP + DEA method is presented as a valuable tool to provide benchmarks for analysts, decision makers at company level, and policy makers in the energy sector, this utility can also be extended beyond the energy sector to other sectors with scattered sources of production, such as mining, transport, or fishing.

## Figures and Tables

**Table 1:** List of case studies currently available in the literature using LC + DEA methods.

Reference	Classification	LC + DEA method	Case study	Key aspects
[38]	Environmental	3-step LCA + DEA	Electronic devices	Comparative ecoefficiency analysis Ecopoint LCA scores used as inputs in the DEA matrix

[29]	Environmental	5-step LCA + DEA	Mussel rafts	Direct link between operational and environmental efficiency Lower economic expenses and lower environmental impacts

[26]	Environmental	3-step LCA + DEA	Trawling vessels in Galicia (NW Spain)	Presentation of the 5-step LCA + DEA method Inclusion of discards as bad output in DEA model

[31]	Environmental	3-step LCA + DEA	Mussel rafts	Joint reduction targets computed for operational inputs and environmental impacts

[32]	Environmental	5-step LCA + DEA	Dairy farms in Galicia (NW Spain)	Benchmarking of environmental impacts Superefficiency calculation for best-performing farms

[39]	Environmental	3-step LCA + DEA	Electric and electronic products	Damage indicators provided by Ecoindicator 99 are included as inputs in the DEA matrix

[40]	Environmental	3-step LCA + DEA	Mahón cheese production (Balearic Islands, Spain)	Analysis of most ecoefficient production techniques Monte Carlo simulations to detect changes in the ecoefficiency ratio due to price fluctuations

[36]	Environmental	5 -step LCA + DEA	Galician fishing fleets divided by gear type and fishing zone	Intra- and interassessment of fishing fleets Operational inputs with low environmental contributions may still imply important economic savings if minimised

[33]	Environmental	5 -step LCA + DEA	Viticulture in the *Rías Baixas *appellation (Galicia, Spain)	5-step LCA + DEA method including superefficiency analysis to identify best-performers Environmental impacts include USEtox and CML impact categories

[41]	Environmental	3-step LCA + DEA	Ecoefficiency of construction materials	DEA is used to rank material alternatives, while LCA is used to quantify the environmental impacts

[42]	Environmental	3-step LCA + DEA	Swiss dairy farms in the Alpine area	DEA matrix made up of environmental impacts as inputs exclusively Description of the relationship between economic and environmental performance

[45]	Environmental	EIO-LCA + DEA	US manufacturing sector	Hierarchical EIO-LCA + DEA method Food, beverages, tobacco, and petroleum identified as drivers of overall environmental impact

[34]	Environmental	5-step LCA + DEA	Soybean farms in Iran	Identification of bad operational practices and recommendation of improvement actions

[27]	Environmental	5-step LCA + DEA	25 wind farms located in southern Spain	Environmental benchmarks for end-of-life applications

[30]	Environmental	5-step LCA + DEA	Galician fishing fleets	Social indicators (e.g., working hours or crew size) included as inputs in LCA + DEA Discussion of the advantages and drawbacks of these inclusions

[28]	Energy	Em + DEA	25 wind farms located in southern Spain	Energy-based ecoefficiency methods Em + DEA viewed as an ecocentric perspective rather than an anthropogenic approach represented by CED and CExD
	Energy	CED + DEA		
	Energy	CExD + DEA		

[37]	Environmental	5-step LCA + DEA	Peruvian *anchoveta* fishing fleet segments	Fishing fleet segments as DMUs rather than individual vessels Clustering of LCI items as inputs in the DEA matrix

[35]	Environmental	5-step LCA + DEA	Rice paddy fields	Distinction between spring/summer rice paddy Superefficiency analysis to identify best-performing entities

**Table 2:** Strengths and weaknesses of implementing the CFP + DEA method.

Aspect	Rating	Justification
Strengths		
Consistency	+	Independency of operational inputs
Quantification	++	This method allows the quantification of the minimisation of operational inputs to attain target efficiency levels
Benchmarking	++	Useful mechanism to determine target environmental improvements for industries and governments
Revision of reference values	+	Environmental benchmarking to recalculate pollutant reference values
Communication	++	Advantages of communicating to stakeholders and general public as compared to LCA + DEA methods due to broader appeal of CFP
Interpretation	+	Reduced complexity as compared to the LCA + DEA methods for knowledge transfer and decision making
Weaknesses		
Factors influencing inefficiency	−−	Lack of identification of the underlying factors of inefficiency
Economic costs	−−	The method does not provide a direct quantification of the costs derived from optimisation procedures
Dependency on sample size	−	The number of DMUs condition the amount of operational items (inputs and outputs) that can be included in the DEA matrix

“++” = major strength; “+” = minor strength; “−” = minor constraint; “−−” = major constraint.
